# *Pumilio-2* Function in the Mouse Nervous System

**DOI:** 10.1371/journal.pone.0025932

**Published:** 2011-10-07

**Authors:** Henrike Siemen, Damien Colas, H. Craig Heller, Oliver Brüstle, Renee A. Reijo Pera

**Affiliations:** 1 Institute for Stem Cell Biology and Regenerative Medicine, Department of Obstetrics and Gynecology, Stanford University School of Medicine, Stanford, California, United States of America; 2 Institute of Reconstructive Neurobiology, Life & Brain Center, University of Bonn, Bonn, Germany; 3 Department of Biology, Stanford University, Stanford, California, United States of America; University of Chicago, United States of America

## Abstract

Coordinated mRNA translation at the synapse is increasingly recognized as a critical mechanism for neuronal regulation. Pumilio, a translational regulator, is known to be involved in neuronal homeostasis and memory formation in Drosophila. Most recently, the mammalian Pumilio homolog *Pumilio-2* (*Pum2*) has been found to play a role in the mammalian nervous system, in particular in regulating morphology, arborization and excitability of neuronal dendrites, *in vitro*. However, the role of *Pum2 in vivo* remains unclear. Here, we report our investigation of the functional and molecular consequences of *Pum2* disruption in vivo using an array of neurophysiology, behavioral and gene expression profiling techniques. We used *Pum2*-deficient mice to monitor *in vivo* brain activity using EEG and to study behavior traits, including memory, locomotor activity and nesting capacities. Because of the suspected role of *Pum2* in neuronal excitability, we also examined the susceptibility to seizure induction. Finally, we used a quantitative gene expression profiling assay to identify key molecular partners of *Pum2*. We found that *Pum2*-deficient mice have abnormal behavioral strategies in spatial and object memory test. Additionally, *Pum2* deficiency is associated with increased locomotor activity and decreased body weight. We also observed environmentally-induced impairment in nesting behavior. Most importantly, *Pum2*-deficient mice showed spontaneous EEG abnormalities and had lower seizure thresholds using a convulsing dosage of pentylenetetrazole. Finally, some genes, including neuronal ion channels, were differentially expressed in the hippocampus of *Pum2*-deficient mice. These findings demonstrate that *Pum2* serves key functions in the adult mammalian central nervous system encompassing neuronal excitability and behavioral response to environmental challenges.

## Introduction

The mammalian *Pumilio-2 (Pum2)* gene is a member of the *Pumilio* gene family that encodes RNA-binding proteins that act as translational regulators. Proteins in the Pumilio family are characterized by their highly conserved RNA-binding domain, the so-called Pumilio homology domain or PumHD, and are known to play an important role in embryonic patterning [1], germ cell maintenance [2] and neuronal functioning in worms and flies [3]. In *Drosophila*, studies focused on the *pumilio* allele *bemused (bem)* demonstrated that *pumilio* is required to maintain neuronal excitability [4] and synaptic development at the neuromuscular junction where it might regulate the translation initiation factor eIF-4E [5] or other potential targets such as the voltage-gated sodium channel *para* [6]. Results suggested that Pumilio may repress *para* translation directly. Even though the repression of *para* also required the cofactors Nanos and Brat, Pumilio was both necessary and sufficient to produce a decrease in sodium current, resulting in decreased membrane excitability. Concomitantly, Pumilio also repressed Nanos mRNA and thus prevented excessive repression of *para* mRNA. Muraro and colleagues also demonstrated that Pumilio affects the fast potassium current, presumably by regulating the voltage-gated potassium channel *Shal* gene [6]. However, there is no evidence that Pumilio protein directly binds to *Shal*. Instead, it was suggested that Pumilio-mediated *para*-repression causes a decrease in S*hal* mRNA and thus, reduced potassium current. Additional studies have also suggested a role for *pumilio* in long-term memory [7], [8]. In one, Dubnau *et al.* identified *pumilio* as an important player in *Drosophila* long-term memory, together with six other genes, all known for regulating mRNA translation or localization [8]. The authors found that expression of *pumilio* is upregulated during long-term memory training and that mutants with a P element insertion in the *pumilio* gene have defective long-term memory.

Despite numerous studies of *pumilio* in lower organisms, relatively little is known about *Pum2* function in mammals. A three-hybrid screen showed that human PUM2 protein binds at least 60 mRNAs *in vitro* [9], several of which are associated with neuronal function. Among these are RACK1 (involved in neuronal excitation), TSC1 (control of axon formation, epilepsy), APP (seizure susceptibility), HSPBAP1 (upregulated in epilepsy patients) and p190 RhoGAPs (involved in neuronal differentiation and process outgrowth). In addition, based on a genome-wide microarray (RIP-Chip) analysis of Pumilio-associated mRNAs in HeLa cells, it was demonstrated that Pum1- and Pum2-associated mRNAs form overlapping sets [10]. Therefore, it might be expected that the *Pum1* and *Pum2* genes are functionally redundant, thus explaining the viability and fertility of *Pum2*-deficient mice [11]. However, examination of the set of mRNAs identified indicated that a subset of mRNAs was associated with either Pum1 or Pum2 proteins, but not both. Notably, the mRNAs that were enriched in the Pum2-associated set included a group of genes linked to Parkinson's disease. Whether Pum2 has additional mRNA targets associated with other neuronal processes or neurodegeneration remains to be determined.

Although it is known that Pumilio proteins mediate translational repression in neurons, the mechanism of repression is unclear. Diverse neuronal functions depend on rapid changes in protein levels, including the constant renewal and turnover of neurotransmitters, receptors and channel proteins that regulate excitability and signal transduction. The majority of proteins involved in these pathways are located in the cell membrane of the neuronal processes and thus, far away from the cell soma. In order to overcome this physical separation and to guarantee synaptic plasticity, neurons have developed a highly elaborated system for translational control of mRNA in axons and dendrites (reviewed in [12]). Indeed, immunostaining of rat hippocampal neurons revealed that Pum2 protein expression is localized primarily in the somatodendritic compartment and often adjacent to postsynaptic PSD95-positive puncta along the dendritic shaft [13]. Moreover, Pum2 protein was also observed in dendritic ribonucleoproteins (RNPs) and stress granules (SGs) of rats and co-localized with the known SG components, TIAR, eIF4E and FMRP. Recently it was shown that, analogous to *Drosophila*, loss of *Pum2* function in rat hippocampal neurons leads to an increase in dendritic outgrowth [14], and that in primary rat neurons Pum2 translation is regulated by the brain-specific dendritic microRNA (miRNA) miR-134 [15].

Previously, our group reported the generation of *Pum2*-deficient mice and characterization of viability and reproductive phenotypes [11]. No phenotypes were observed in the females, while the males displayed a subtle phenotype (smaller testes). In the present study, we describe unexpected phenotypes that were uncovered in analysis of the nervous system in *Pum2*-deficient mice. We performed behavioral, electroencephalographic (EEG) and molecular analysis and discovered that *Pum2*-deficiency leads to abnormal behaviors and cortical excitability.

## Results

### Characterization of *Pum2* expression in the brain of *Pum2^XE772^* mice

The transgenic *Pum2^XE772^* line carries a gene trap mutation inserted between exon 10–11 that results in a truncated Pum2 protein lacking the Pum-HD [11]. Viability and germ line phenotypes of *Pum2^XE772^* homozygous mice are well characterized; however, the effects of the loss-of-function of *Pum2* on the nervous system have not been examined. Histological examination of brain tissues (via standard hematoxylin-eosin staining and immunohistochemistry of NeuN (Neuronal Nuclei), Neurofilament and GFAP (Glial Fibrillary Acidic Protein)) did not reveal any abnormal morphological or developmental defects in *Pum2^XE772^* homozygous mice (Fig. S1).

**Figure 1:**
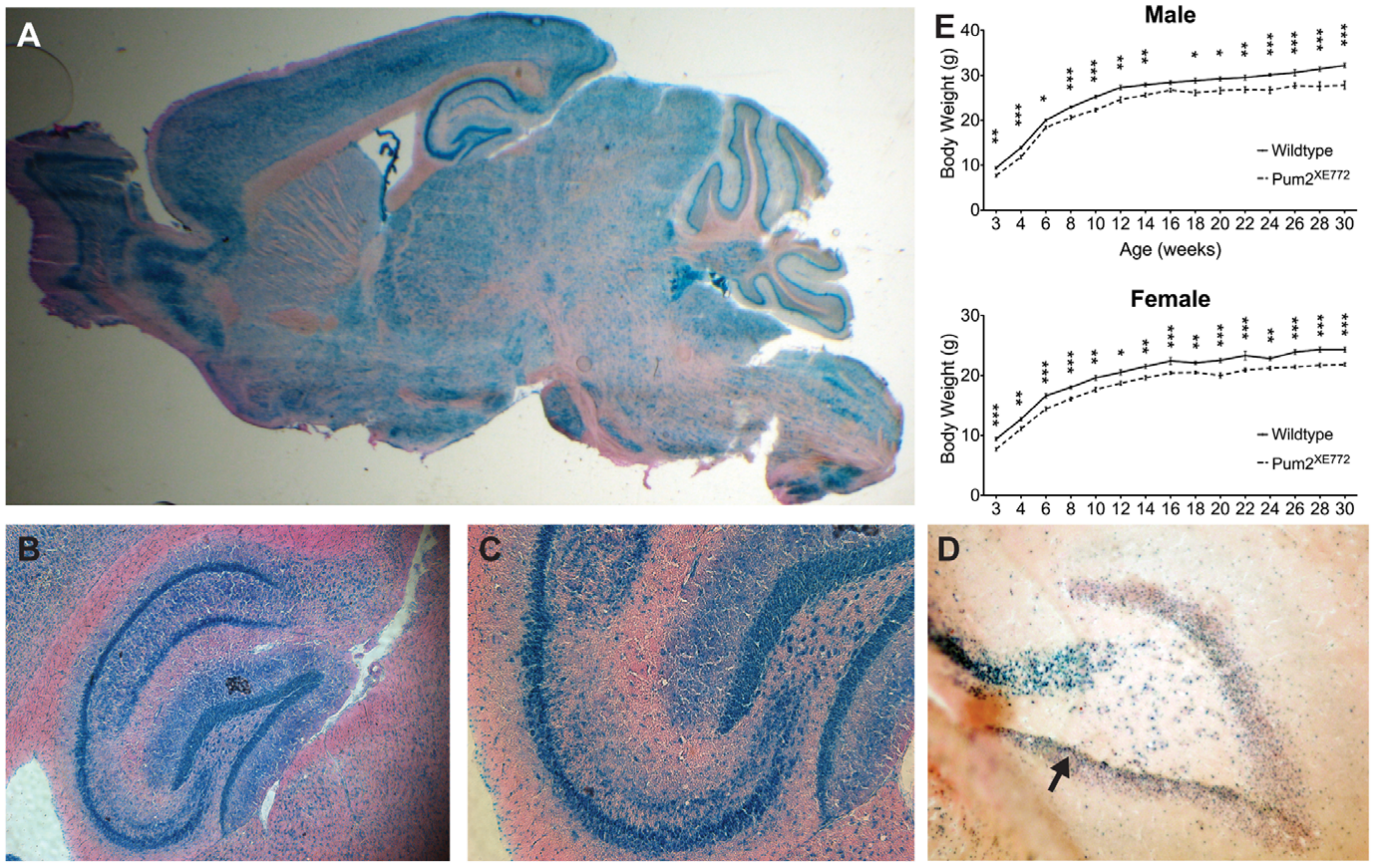
*LacZ* reporter expression and body weight phenotypes. (A) The *LacZ* reporter is expressed in the brain of *Pum2^XE772^* mice. (B, C) Expression is strong in the hippocampus. (D) In several mice we could detect noticeably strong Pum2 expression in the subgranular zone (SGZ) of the dentate gyrus (arrow). (E) Body weight of *Pum2^XE772^* mice and wildtype littermates. *Pum2*-deficient male (top) and female mice (bottom) have significantly lower body weight than wildtype littermates at all ages between 3 and 30 weeks (Mean ± SEM, *p<0.05, **p<0.01, ***p<0.001).

The LacZ reporter in the gene trap allele allows for the visualization of *Pum2* reporter expression in mouse tissue via enzymatic staining assays. We observed strong LacZ staining throughout the whole brain, indicating high expression of *Pum2* (Fig. 1, A–D). In the hippocampus of some mice, Pum2 is expressed at higher levels, although not exclusively, in the subgranular zone (SGZ) of the dentate gyrus (Fig. 1D, arrow).

### *Pum2^XE772^* mice have a lower body weight than control littermates

We observed that *Pum2^XE772^*-homozygous mice were noticeably smaller than their wildtype and heterozygous littermates. To quantify potential differences in size, the body weight of mice of all three genotypes was recorded from the time of weaning for a period of 30 weeks (Fig. 1E). On average, *Pum2^XE772^* mice weighed 1.7–4.4 g less than the control littermates. This difference was very significant overall (p<0.01) and highly significant for most time points (p<0.001), in both males and females. Moreover, the average body weight of heterozygous *Pum2^XE772^* mice was between that of homozygous and wildtype mice, suggesting a dosage effect of *Pum2*.

### *Pum2^XE772^* mice are hyperactive

**Figure 2:**
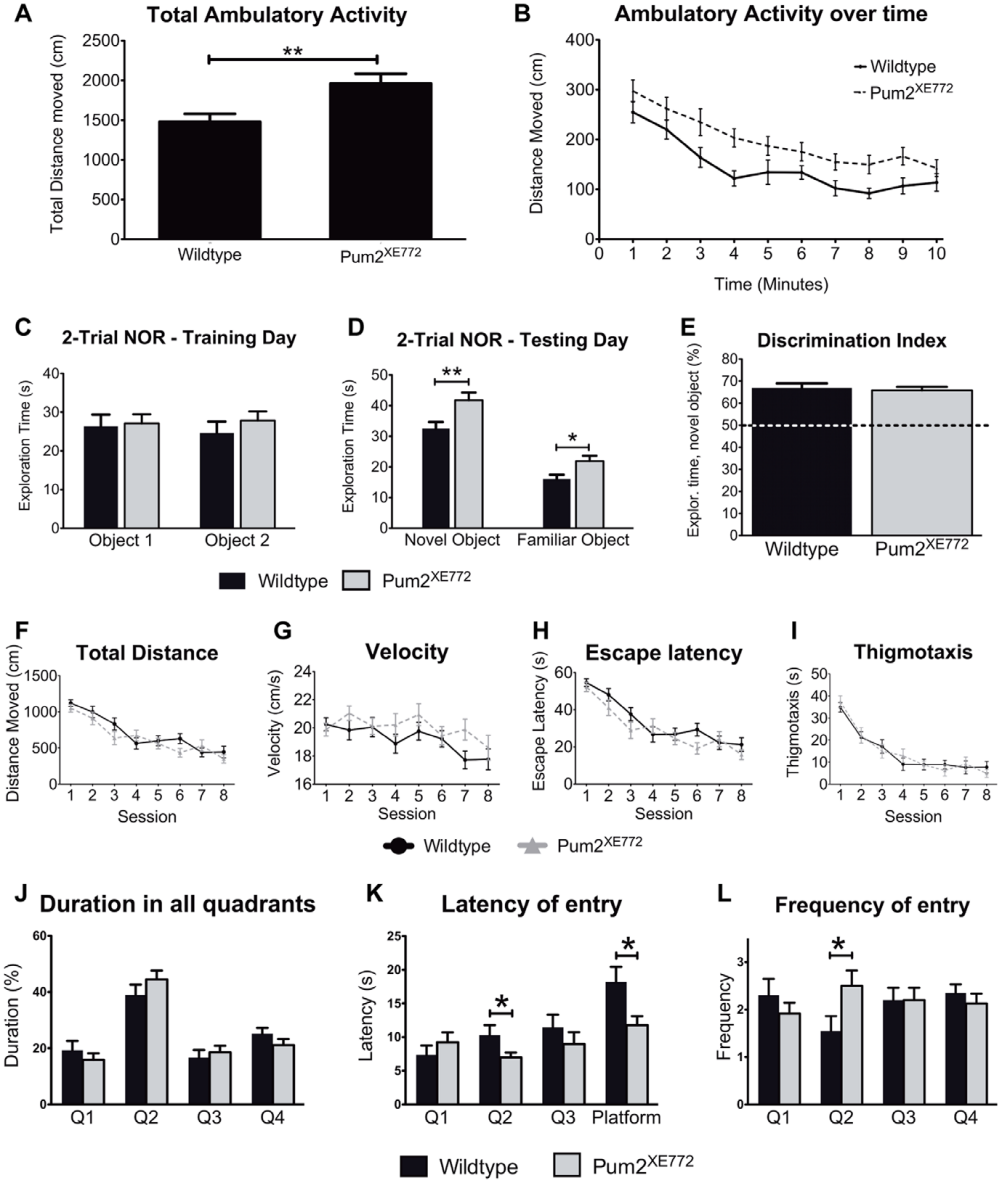
Behavioral tests of *Pum2^XE772^* and wildtype mice. (A–B) Activity chamber. (A) *Pum2^XE772^* mice travel an overall longer distance compared to wildtype mice. (B) Mice of both genotypes show the same adaption pattern to the new environment (less activity over time). (C–E) Novel object recognition test (NOR). (C) During training sessions, wildtype and *Pum2^XE772^* mice spend equal time exploring both objects. (D) Mice of both genotypes memorize a known object and spend more time with a novel object. *Pum2^XE772^* mice spend significantly more time exploring both objects than wildtype mice do. (E) The discrimination index shows that mice of both genotypes recognize the novel object. (F–I) Morris water maze - Training sessions. Mice were trained to find the platform in 8 sessions over 4 days. Measurement of basic locomotor parameters confirmed a learning curve in both genotypes: (F) Total distance each group swam to reach the platform, (G) velocity during trials, (H) time needed to find the platform and (I) thigmotaxis (Mean ± SEM). (J–L) Morris water maze - Probe trial. Subsequent to the training, the platform, originally located in Q2 (target quadrant), was removed and movement of the mice during four 30-second trials was recorded. *Pum2^XE772^* mice have a significantly higher frequency and lower latency of entry in the target quadrant. (J) Percentage of total time the mice spend in each quadrant (Q1–Q4). (K) Latency of entering each quadrant (Q1–Q3, mouse was placed into Q4) and the actual location where the platform was located within target quadrant Q2 (Platform). (L) Frequency of entry into each quadrant (Mean ± SEM, *p<0.05, **p<0.01).

To determine whether *Pum2^XE772^* mice exhibit behavioral abnormalities, we performed a set of behavioral tests. All tests were performed with males only. Initial control tests confirmed that the mice had normal vision, hearing, and olfactory ability. In addition, overall locomotor activity of the mice was recorded in an activity chamber. An infrared detector system tracked distance moved, velocity, resting time and the time spent in defined areas during a 10-minute period. While there were no differences in average velocity or jump count, there was a significant difference in the total distance moved between *Pum2^XE772^* and wildtype mice (Fig. 2, A–B). Wildtype mice moved 1479±99 cm (N = 22) in total whereas *Pum2^XE772^* mice traveled 1961±123 cm (N = 25; *p*<0.01) on average during a 10-minute period. Both genotypes moved less over time, indicating a habituation or learning effect with no significant difference between the two curves (Fig. 2B).

Next, the Open Field test was used to examine exploration habits and anxiety levels by recording the time spent in different zones within a large cage. All mice regardless of genotype spent the majority of time in the border areas of the cage and avoided the center of the open field; no differences in anxiety related parameters were detected between the groups (Fig. S2). However, in agreement with the activity chamber result, *Pum2^XE772^* mice traveled an overall significantly greater distance than wildtype mice (6469±440 cm vs. 5037±557 cm; N = 7,6; *p*<0.05). Also, *Pum2^XE772^* mice displayed a significantly higher velocity than wildtype mice (10.78±0.73 cm/s vs. 8.4±0.5 cm/s; N = 7, 6; *p*<0.05).

### *Pum2^XE772^* mice show a different behavioral strategy in memory tests

Given that *Pumilio* is involved in the formation of long-time memory in flies, our next aim was to focus on the behavioral assessment of memory performance We used the non-stressful Novel Object Recognition (NOR) test to examine the spontaneous ability of the mice to recognize a novel object in a familiar environment. In training sessions, mice were placed in an open field with two objects and the time spent with each object was recorded. For the 2-trial test, mice were first exposed to two objects for 10 minutes. After 24 hours the mice were returned to the open field and presented with one familiar object from the first trial and one novel object. Throughout the training, mice of both genotypes spent roughly the same amount of time exploring each object (35–45 seconds, Fig. 2, C). During the test trial, both wildtype and *Pum2^XE772^* mice spent more time with the novel object (discrimination index of higher than 50%), indicating that they remembered the known object and had normal learning abilities (Fig. 2, D–E). Interestingly, we found that Pum2^XE772^ mice spend significantly more time with both objects than wildtype and heterozygous littermates (Fig. 2, D). However, the discrimination index was not significantly different (wildtype: 66.9% (±2.1) vs. *Pum2^XE772^*: 65.8% (±1.6)). We repeated the task as a 5-trial test to allow for improved habituation. Mice were exposed to the same two objects four times for 5 minutes each, with a 3 minute interval between each exposure. Twenty-four hours later, a fifth trial was performed, with one object being replaced by a novel object. Consistent with the 2-trial test, mice of both genotypes spent more time exploring the novel object. Again, *Pum2^XE772^* spent more time overall with both objects, though the discrimination index was similar to wildtype mice (Fig. S3).

To further assess spatial learning and memory, we used the Morris water maze. Initially, the mice were trained for 4 days (24 trials in 8 sessions) to find a hidden platform in the pool. During the training, several factors to determine a learning curve were assessed, including escape latency, total distance traveled, thigmotaxis and velocity. There was no significant difference in these parameters between genotypes and therefore, we assumed that hyperactivity did not play a role. In addition, we observed a clear learning curve with a decrease in escape latency and total distance moved in both genotypes (Fig. 2, F–I; Table 1). Following the training, a probe trial was performed by removing the platform. Both wildtype and *Pum2^XE772^* mice were able to locate the target quadrant during the 30 second long trial, indicating normal spatial memory (Fig. 2, J). However, *Pum2^XE772^* mice had significant shorter latency to first entry of the target quadrant compared to wildtype littermates (Fig. 2, K). This was even more pronounced in the latency to enter the area where the platform was located during the training. Also, *Pum2^XE772^* mice entered the target quadrant more frequently (Fig. 2, L).

**Table 1:** Morris Water Maze.

	Wildtype	Heterozygous	*Pum2^XE772^*
**Training**			
***Total distance traveled (cm)***	5619 (±274.5)	5264 (±258.9)	5141 (±248.7)
*Session 1*	1119 (±44.2)	1049 (±88.9)	1049 (±53.1)
*Session 8*	443.2 (±79.3)	310.6 (±52.5)	350 (±57)
***Escape latency (s)***			
*Session 1*	54.6 (±2)	50.43 (±3.4)	52.29 (±2.6)
*Session 8*	21.24 (±3.7)	13.71 (±2.33)	15.84 (±2.74)
***Thigmotaxis (s)***			
*Session 1*	34.87 (±2.2)	34.42 (±3)	37.41 (±2.7)
*Session 8*	7.67 (±2.6)	3.31 (±1.2)	4.89 (±1.8)
***Velocity (cm/s)***	19.19 (±0.43)	20.58 (±0.78)	20.01 (±0.53)
**Probe Trial (Test day)**			
*Total distance traveled*	660 (±22.9)	714.4 (±22.2)	708.1 (±15.4)
*Velocity*	22.1 (±0.8)	23.9 (±0.7)	23.7 (±0.5)

Shown are values ±SEM; N = 17 (wt), 12 (heterozygous), 19 (*Pum2^XE772^*).

### Nesting behavior is disturbed in *Pum2^XE772^* mice

**Figure 3:**
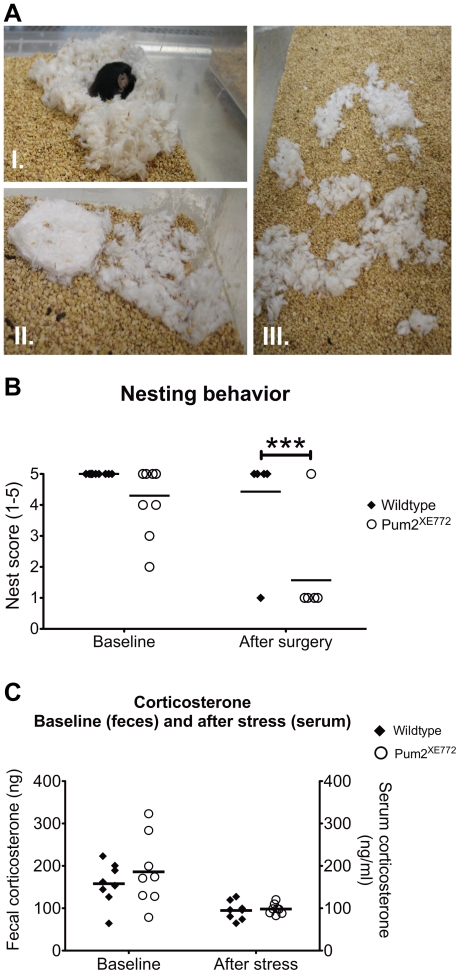
Nesting behavior and stress. (A–B) Nesting behavior. Nestlets were provided at night and nests were scored the next morning. (A) Examples for nest scoring: top left picture show score 5 (perfect) nest with walls higher than the mouse body for more than 50% of its circumference, bottom left picture shows score 2: Nestlet is only partially torn (50–90%), right hand picture shows score 3: less than 90% of the Nestlet is within a quarter of the cage floor area. (B) *Pum2^XE772^* mice have impaired nest building abilities. The baseline effect is further (significantly) enhanced after surgery. (C) Corticosterone analysis in baseline (from feces) and after stress (serum). For baseline measurements, total corticosterone was extracted from feces collected from an 8-hour period. To analyze corticosterone levels under stressed, mice were restrained for 20 minutes and immediately sacrificed to collect blood. There was no significant difference between *Pum2^XE772^* mice and wildtype littermates under either condition (***p<0.001).

When provided with nesting material, most laboratory mice of all common strains will build nests that can subsequently be qualitatively scored. Nesting capacity in mice is a sensitive indicator of general health status as well as brain function (in particular hippocampus dependent functionalities) [16], [17]. In baseline conditions, 10 male *Pum2^XE772^* mice and 10 wildtype littermates were placed in single housing and provided with Nestlets an hour before dark. The next morning, nests were scored on a scale of 1 to 5 (5 being the best) and the remaining nesting material was weighed. While all wildtype mice built near perfect nests with more than 50% of the nest wall being higher than the mouse body height, only 60% of the *Pum2^XE772^* mice built 5-score nests and the other 40% did not shred the Nestlet completely or made flat nests without walls (Fig. 3, A–B). Interestingly, while this baseline scoring already showed a trend that the nest building ability is impaired in *Pum2^XE772^* mice, the effect was much more pronounced in animals that underwent surgery for EEG recordings. After surgery, mice were housed together for 7 days and then moved to separate cages for recordings. The mice were adjusted to recording cages for one week without cables attached to the implant. After one week, mice were provided with a Nestlet and nests were scored after 12 hours as previously. Of the 8 wildtype mice, 7 built a perfect or near perfect (score 5) nest and one mouse did not shred the Nestlet at all. In contrast, 7 out of 8 *Pum2^XE772^* mice did not touch the Nestlet and only one mouse built a complete 5-score nest. This failure indicates that an innate nesting deficit appeared to be aggravated in *Pum2^XE772^* during the recovery period after significant pertubation.

### Corticosterone levels are similar in wildtype and *Pum2^XE772^* mice under stressed and non-stressed conditions

Because the aforementioned deficient behaviors occurred partly in response to environmental changes and perturbances, we next sought to assess the levels of the stress hormone corticosterone (CORT), since an abnormal stress response could account for such changes. Baseline CORT levels were determined by extraction from feces collected over a period of 16 hours. On average, CORT metabolite levels were the same in wildtype (282±78 ng/g feces, N = 8) and *Pum2^XE772^* mice (287±108 ng/g feces, N = 8) under unstressed conditions (Fig. 3, C). We then compared CORT levels in mice after stress induced by restraining mice for 20 minutes immediately followed by blood collection. Again, levels of CORT metabolites were similar in *Pum2^XE772^* mice (98.2±12.3 ng/ml serum, N = 8) and wildtype littermates (94.8±21.5 ng/ml serum, N = 8), indicating that CORT levels, a sensitive measure of the stress component, are not altered in *Pum2^XE772^* mice.

### A subset of *Pum2^XE772^* mice develop spontaneous seizures

We observed that some *Pum2^XE772^* mice developed seizures that were induced by handling. These seizures were observed only in *Pum2^XE772^* animals and only in those that were older than 5 months. The seizures were induced by simply moving the cage or handling the mouse. The types of seizures ranged from small seizures that included stiffening of the body and a curling of the tail, sometimes accompanied by foaming at the mouth, to generalized tonic clonic seizures resulting in the mouse falling on its side and undergoing contraction for approximately one minute. Tonic-clonic seizures were observed in a smaller number of *Pum2^XE772^* mice whereas spontaneous seizures in wildtype and heterozygous mice were never detected.

### *Pum2^XE772^* mice have abnormal EEG features and have lower chemically-induced seizure thresholds

**Figure 4:**
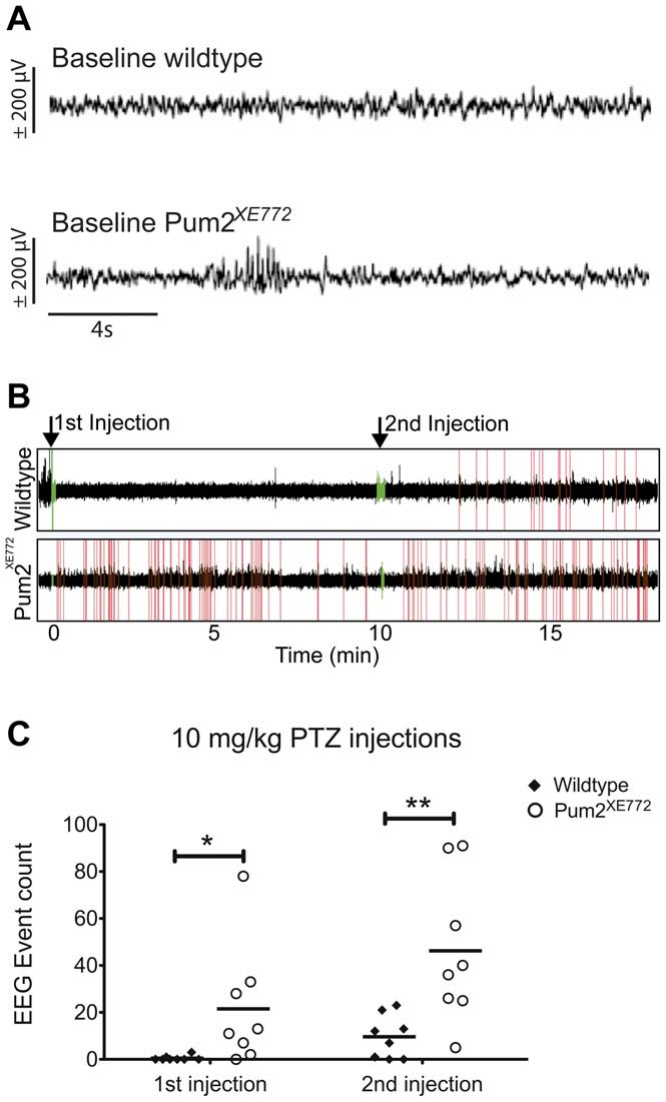
PTZ seizure screen (10 mg/kg bodyweight). (A) Representative EEG samples in baseline in a wildtype (top) and a *Pum2^XE772^* mouse (bottom). Traces represent 20 seconds of EEG recordings (200 Hz sampling rate, 0.7–50 Hz band filtering). Abnormal EEG events, characterized by short lasting (2 seconds) 5–7 Hz bursts, are seen in 50% of KO mice and never occur in WT mice. (B) Representative example of an event count for one wildtype (top) and one *Pum2^XE772^* mouse (bottom) after injection of 10 mg/kg PTZ. Injection times are marked with green (arrows), counted events in red. (C) *Pum2^XE772^* mice have significantly higher numbers of EEG events both after the first injection of 10 mg/kg PTZ and after a second injection 15 minutes later (*p<0.05, **p<0.01).

To quantify spontaneous seizures and to examine seizure susceptibility in *Pum2^XE772^* mice, we performed a series of EEG recordings with concomitant video analysis. Baseline recordings of untreated *Pum2^XE772^* mice and wildtype littermates revealed that 50% of the *Pum2^XE772^* mice show spontaneous EEG abnormalities (5 Hz high amplitude trains) that are accompanied by typical signs of behavioral pausing, reminiscent of absence seizure (Fig. 4, A). Next, we tested whether *Pum2^XE772^* mice are more susceptible to the seizure-inducing drug, pentylenetetrazole (PTZ) [18]. Mice with low seizure thresholds are usually studied using a dose of approximately 45 mg/kg PTZ, but because of the baseline phenotype and prior pilot studies, we chose to initially study the effects of a lower dosage of PTZ. Thus, we injected mice with a below-threshold dose of 10 mg/kg PTZ. Simultaneous EEG recordings revealed that the PTZ injections elicited abnormal EEG spikes similar to the ones seen in baseline (5 Hz trains) in 6 of 8 *Pum2^XE772^* mice (Fig. 4, B–C, Table 2). As in baseline, the abnormal EEG features were accompanied with behavioral pausing. In contrast, only 2 of 8 wildtype littermates showed mild EEG abnormalities after PTZ injection and these were less frequent and of lower intensity than those in *Pum2^XE772^* mice. Fifteen minutes after the initial injection, all mice were injected with a second dose of 10 mg/kg PTZ, which induced EEG spikes in all mice but one wildtype mouse. However, the number of EEG spikes was significantly higher in Pum2^XE772^ mice (Fig. 4, C, Table 2).

**Table 2:** Results of 10 mg/kg PTZ screen.

	Mean (±SEM)		*p*-Value	N
	Wildtype	*Pum2^XE772^*		
***10 mg/kg***				
*Event count after 1^st^ injection*	0.5±0.38	21.5±9.07	*0.0052* **	8; 8
*Event count after 2^nd^ injection*	9.63±3.25	46.25±10.97	*0.0054* **	8; 8
***45 mg/kg***				
*Time until 1^st^ event (s)*	159.9±42.67	70.00±13.88	*0.0403* *	8; 8
*Time until seizure (s)*	558.6±124.4	265.5±82.73	0.065	8; 8
*Time: 1^st^ spike – seizure (s)*	398.8±92.16	195.5±71.68	*0.03* *	8; 8
*Duration of seizure (behavioral events, s)*	18.72±1.91	45.12±4.8	*0.0079* **	5; 5
*Event count after seizure (3 h)*	57.00±23.48	147.9±36.74	*0.0289* *	8; 7

*p<0.05;

**p<0.01.

**Figure 5:**
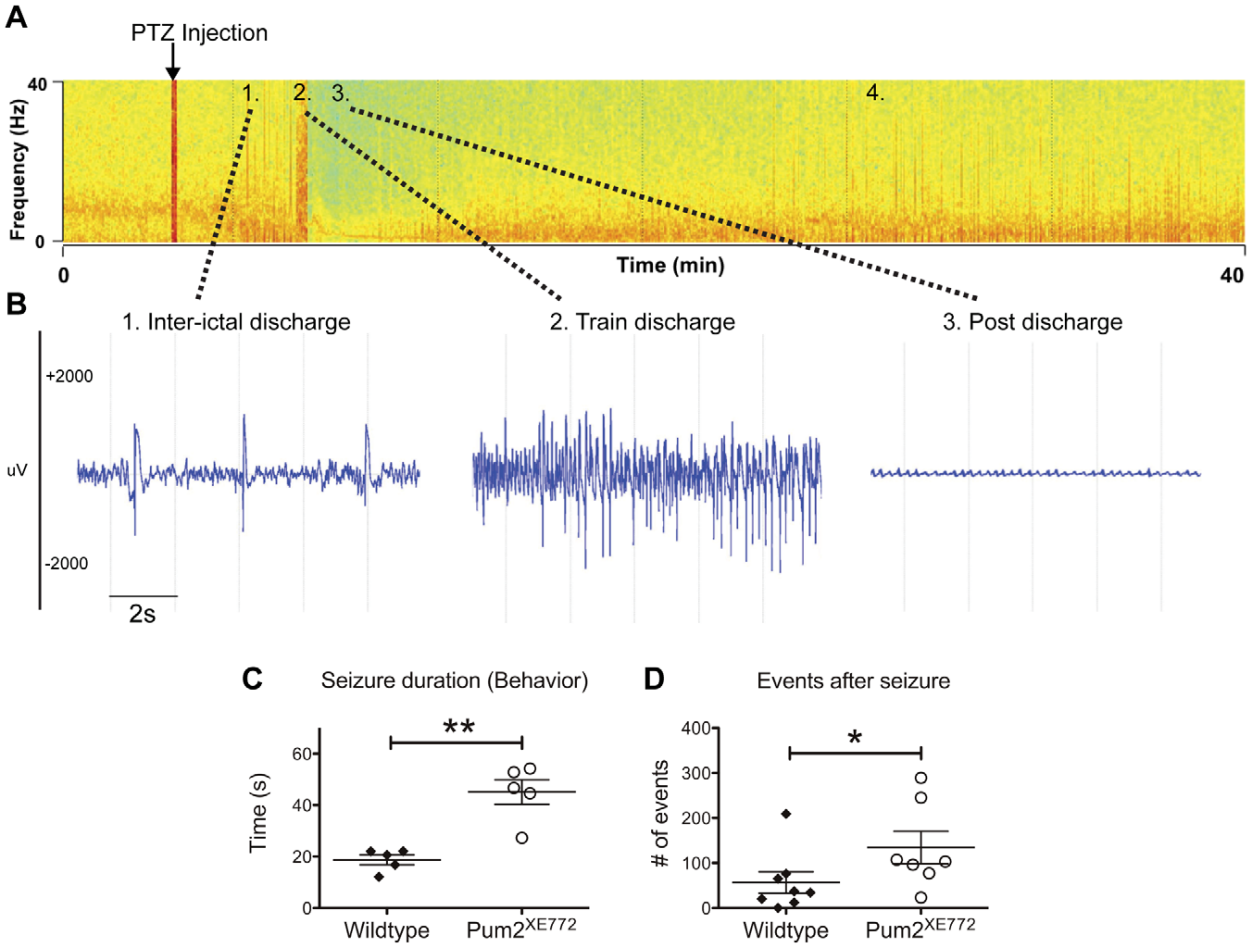
PTZ seizure screen (45 mg/kg bodyweight). (A) Actual color-coded spectral analysis (EEG power vs. frequencies in ordinate and time in abscise) of a *Pum2^XE772^* EEG. Spectral analysis was performed using a FFT (1024 Hz) on 4 seconds epochs and EEG power per frequency bin was subsequently color coded as high in red, intermediate in yellow and low in green, allowing the visualization of abnormal EEG events of high intensity (red lines). A single 45 mg/kg PTZ injection (arrow) induces typical interictal discharges within 15 min (1.), followed by a paroxysmal episode of train discharges (2.) and a post discharge episode characterized by low intensity EEG (postictal period, 3.). After approx. 20 min the EEG is normalized but discharges are still seen for several hours (4.). (B) Actual EEG samples of zone 1. (interictal discharges), 2. (train discharges) and 3. (postictal EEG). Of note, discharges occurring in zone 4. (recovery) are of the same nature as discharges in zone 1. (C) Quantification of behavioral events: *Pum2^XE772^* mice have significantly longer tremor duration (body twitches and jerks) during the postictal period compared to wildtype mice. Despite a flat EEG, *Pum2^XE772^* mice had increased behavioral manifestations. (D) Quantification of discharges during a 3-hour period after EEG recovery (red lines in A, 4.). *Pum2^XE772^* mice have significantly more abnormal EEG events following the tonic-clonic seizure (*p<0.05, **p<0.01).

After a two-week recovery period, we studied the effects of a circa-threshold PTZ dose of 45 mg/kg. This dosage has been routinely used for inducing generalized seizures that can be scored according to a standardized scale based on behavioral manifestations and EEG [19]. Using this standardized scoring protocol, all but one wildtype mouse had severe seizure episodes resembling clonic-tonic seizures characterized by intense convulsive manifestations, including tremor/jumping associated with high amplitude fast EEG discharges building up to a paroxysmal episode of a train of high amplitude EEG discharges a few minutes after injection (Fig. 5, A–B). One *Pum2^XE772^* mouse had multiple seizures and actually died. Interestingly, the evolution of the seizure like activities (both behavior and EEG) appeared to be different between wildtype and *Pum2^XE772^* mice. When we measured the time to the first epileptic EEG spike after injection and the onset time of the paroxysmal EEG discharges train, we found that *Pum2^XE772^* mice had a shorter latency from the first event to the paroxysmal part of the seizure attack (Table 2). Additionally, while the length of the EEG discharge was approximately the same between wildtype and *Pum2^XE772^* mice, there was a significant difference in the behavior manifestations during the seizure (Movies S1, S2, S3, S4). Both wildtype and *Pum2^XE772^* mice had tonic-clonic events that started with Straub tail and occasionally whole body jerks followed by tonic convulsions (jumping). While seizures in all wildtype mice finished at this point with postictal flaccidity (and EEG flattening, Movies S1, S3), we noticed that all *Pum2^XE772^* mice had an additional onset of positive behavioral features (running and jumping) before becoming postictal (Movies S2, S4). Additionally, the total duration of seizures was significantly longer in *Pum2^XE772^* mice compared to wildtype littermates (Fig. 5, C). Finally, the number of abnormal EEG discharges during a 3-hour period following the injection, was significantly higher in *Pum2^XE772^* mice compared to wildtype littermates (Fig. 5, D). Overall, our data indicates that *Pum2^XE772^* mice have abnormal EEG features in baseline and a lower seizure threshold compared to wildtype mice.

### Gene expression is altered in the hippocampus of *Pum2^XE772^* mice

In attempts to further understand underlying pathways associated with *Pum2* function in the brain, we performed gene expression analysis using quantitative RT-PCR. Since the hippocampus is a known epileptogenic region, we focused the analysis on the three distinct hippocampus regions, i.e., CA1, CA3 and the dentate gyrus of the adult brain. Utilizing a microfluidic qPCR system allowed us to analyze a large variety of genes. We mainly selected genes that are associated either with epilepsy, including ion channels and synaptic genes, or with Pumilio-dependent translational regulation, including mouse orthologs of known Pumilio targets in Drosophila and potential mammalian Pum2 targets (Table S1). We normalized expression data with either a set of three ubiquitous reference genes (Ctnnb1, Gapdh and Ubc) or with a set of three neuron-specific reference genes (Eno2, Syp and Tubb3). After two analyses (low and high amount of RNA template) with 96 Taqman probes we chose 48 genes for further analysis.

Two different Taqman probes were used for the analysis of *Pum2* expression; one that binds within the 5′ region and is expressed in mRNAs from both the *Pum2^XE772^* and wildtype mice, and another probe specific to a region in the 3′ region of the gene, that is deleted in the *Pum2^XE772^* mouse (Pum2-2). Gene expression analysis confirmed the difference between the two probes: We detected only a very low background signal with the Pum2-2 probe in *Pum2^XE772^* tissues. Interestingly, we observed that *Pum2* expression was reduced by approximately 50% in *Pum2^XE772^* mice in comparison to wildtype littermates. We also looked at the expression level of Pum1 but there were no significant changes in the hippocampus.

**Figure 6:**
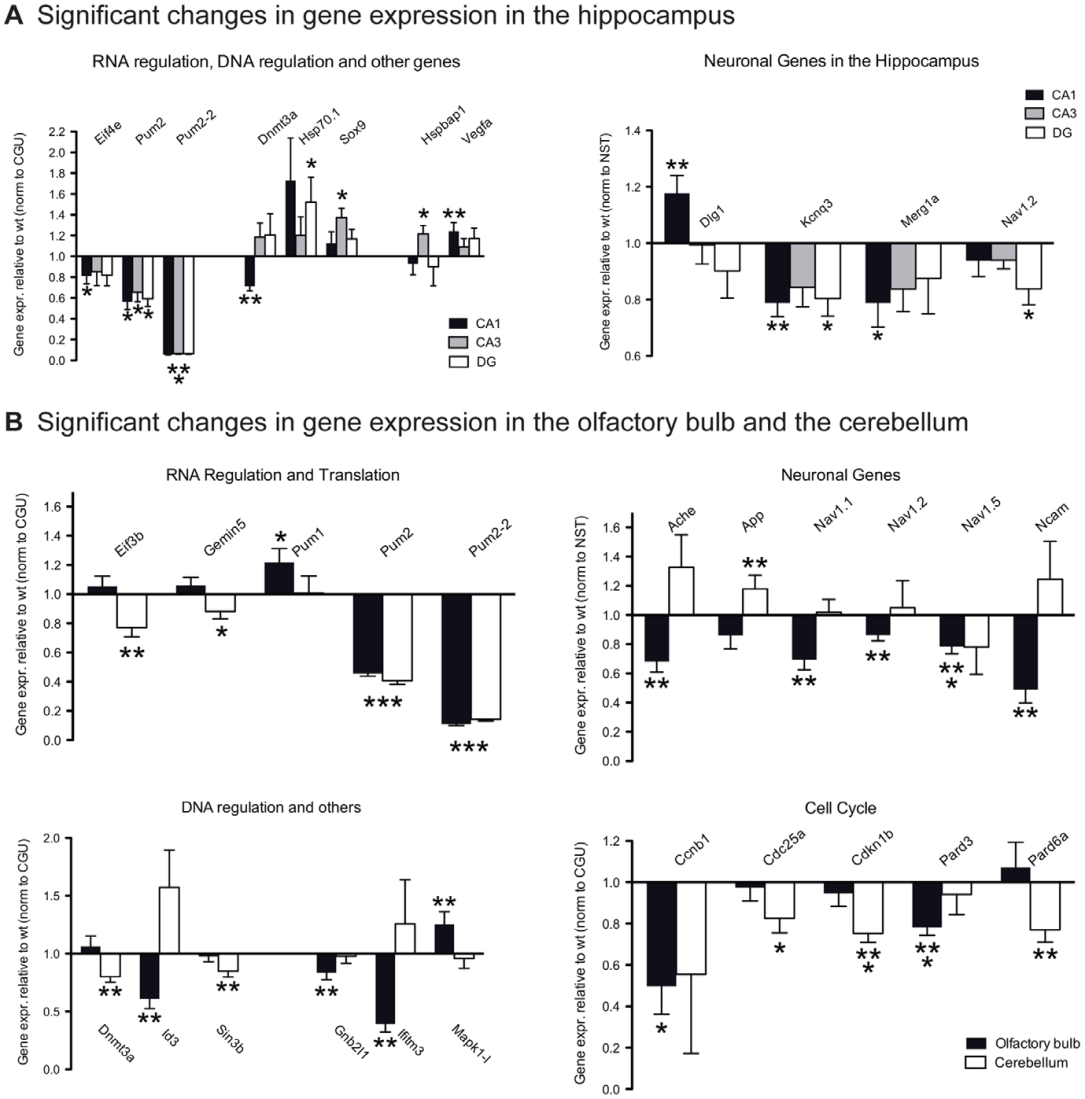
Gene expression in the brain. Data are normalized to the housekeeping genes Ctnnb1, Gapdh and Ubc (CGU) or the neuron-specific genes Eno2, Syp and Tubb3 (NST). The graphs show fold difference of *Pum2^XE772^* tissue compared to wildtype. Plotted are only genes with expression levels significantly different from wildtype tissue (Mean ± SEM, *p<0.05, **p<0.01, ***p<0.001).

We found several genes that were differentially expressed in *Pum2^XE772^* tissue compared to wildtype (Fig. 6, Table 3). However, most of the changes in gene expression, even though statistically significant (*p*<0.05), were relatively small. Of particular note, we observed that the majority of neuronal genes that we analyzed were downregulated in *Pum2^XE772^* tissues compared to wildtype mice. For instance, the potassium channels Kcnq3 and Merg1a were significantly downregulated by approximately 25% in CA1, whereas Dlg1 was significantly upregulated in CA1.

**Table 3:** Gene expression analysis in the hippocampus.

	CA1		CA3		DG	
Gene	Fold expression (*Pum2^XE772^*/wt)	*p*-value	Fold expression (*Pum2^XE772^*/wt)	*p*-value	Fold expression (*Pum2^XE772^*/wt)	*p*-value
*RNA processing and translation (normalized to CGU* 1 *)*						
Eif4e	0.81	***0.035*** *	0.85	0.315	0.82	0.132
Pum2	0.56	***0.001*** *	0.66	***0.012*** *	0.59	***0.001*** *
Pum2-2	0.06	***<0.0001*** ***	0.06	***<0.0001*** ***	0.06	***<0.0001*** ***
*DNA methylation and transcription (normalized to CGU* 1 *)*						
Dnmt3a	0.71	***0.0001*** ***	1.08	0.602	1.21	0.258
Hsp70.1	1.73	0.054	1.20	0.281	1.52	***0.028*** *
Sox9	1.12	0.274	1.28	***0.024*** *	1.17	0.084
*Other (normalized to CGU* 1 *)*						
Hspbap1	0.93	0.508	1.21	***0.012*** *	0.90	0.581
Vegfa	1.24	***0.008*** **	1.09	0.264	1.17	0.092
Dlg3	1.19	0.139	0.95	0.697	0.76	***0.011*** *
Dlg4	0.98	0.793	1.02	0.863	0.87	***0.043*** *
*Neuronal (normalized to NST* 2 *)*						
Dlg1	1.18	***0.007*** **	0.99	0.944	0.90	0.336
Kcnq3	0.79	***0.002*** **	0.84	0.065	0.80	***0.015*** *
Merg1a	0.79	***0.045*** *	0.84	0.091	0.88	0.381
Nav1.2	0.94	0.304	0.94	0.122	0.84	***0.026*** *

1 CGU: Ctnnb1, Gapdh, Ubc;

2 NST: NSE (Eno2), Syp, Tubb3;

*p<0.05;

**p<0.01,

***p<0.001.

The set of genes involved in RNA processing and transcriptional regulation was largely expressed at the same level both in Pum2^XE772^ and in wildtype tissue. Downregulation of Eif4e was significant in CA1; however, the fold change was small (0.8-fold). The differences in gene expression were greater in genes involved in DNA methylation and transcriptional regulation. For instance, we found a 30% decrease in *Dnmt3a* expression in CA1, which was highly significant, and a 1.2–1.7 fold upregulation of *Hsp70.1* in all three tissues. The transcriptional regulators, *Nfat5*, *Sin3b*, *Sox9* and *Stat3* were also all upregulated in *Pum2^XE772^* tissue with the increase of *Sox9* expression in CA3 being statistically very significant (1.4-fold). Expression of the Map kinase genes, *Mapk1* and *Mapk14*, which are potential targets of Pum2-mediated repression, was not significantly altered.

We also examined gene expression levels of the same genes in the olfactory bulb and in the cerebellum. As in other tissues, *Pum2* expression was downregulated in these sites by 50% in *Pum2^XE772^* mice compared to wildtype. Interestingly, expression of the homologue, *Pum 1*, was slightly, but significantly increased in the olfactory bulb. The voltage gated sodium channels, *Nav1.1*, *Nav1.2* and *Nav1.5* were also significantly downregulated in the olfactory bulb, and *Nav1.2* was also decreased in the hippocampus. In addition, the expression of several cell cycle genes (*Ccnb1*, *Cdc25a*, *Cdkn1b*, *Pard3*) was decreased in *Pum2^XE772^* mice both in the olfactory bulb and in the cerebellum, but not in the hippocampus.

## Discussion

The *Pumilio* family of genes encodes RNA regulators that play important roles in the development, including the nervous systems, of flies and worms. Here, we sought to examine *Pumilio* function in the mammalian central nervous system. Our strategy was based on the development of a mouse strain carrying a gene trap allele with a LacZ reporter construct, the *Pum2^XE772^* strain. We observed several notable findings that have not previously been described. First, we observed that *Pum2* is highly expressed in cortical areas of the brain, the hippocampus and to a lesser extent in other regions such as hypothalamus that could account, in part, for the behavioral and EEG changes observed. Second, we observed that *Pum2^XE772^* mice are characterized by key behavioral deficiencies, which relate to discrete misadaptations to challenging environmental circumstances and abnormal cortical excitability. Third, we observed widespread changes in mRNA expression in *Pum2^XE772^* mice relative to wildtype.

The *Pumilio* family of genes has been reported to play a role in stem cells in invertebrates. In this respect, our finding of high expression of Pum2 reporter in the SGZ of the dentate gyrus in *Pum2^XE772^* mice is interesting. The SGZ and the olfactory bulb are two regions of adult neurogenesis and contain proliferating neural progenitor/stem cells [20], [21]. *Pumilio* is also required for germ line stem cell maintenance in *Drosophila* and the worm, *Caenorhabditis elegans*, and for planarian stem cell self-renewal [22]. Thus, it has been suggested that Pumilio has an ancestral function in stem cell maintenance. However, when we generated several embryonic stem cell lines from *Pum2^XE772^* mice, we did not readily observe defects in self-renewal in these lines. We also were able to differentiate these *Pum2*-deficient ES cells into neural precursor cells according to an established protocol [23] and did not notice any differences in self-renewal or differentiation potential in these lines compared to wildtype lines at a qualitative level. Therefore, it is likely that *Pum2* is either not involved in the maintenance of neural stem cells or is dispensable, possibly because the two genes, *Pum1* and *Pum2*, may have overlapping functions. Nevertheless, it will be of interest to further analyze neurogenesis, neural stem cell function and migration in *Pum2^XE772^* mice.

The finding that *Pum2^XE772^* mice have a lower body weight might be correlated with the observation that they are hyperactive and spend significantly more time moving than wildtype mice. Changes in locomotor activity are a common syndrome in transgenic mice. It is estimated that 1.5% of all genes in the mouse genome can cause hyperactivity when disrupted [24], many of which are neurotransmitters and ion channels. Hence, it is difficult to pinpoint the cause of increased locomotor activity in *Pum2^XE772^* mice. Hyperactivity observed in *Pum2^XE772^* mice can also be interpreted as an increased exploration time, which is in accordance with the results of the NOR test. One hypothesis is that *Pum2^XE772^* mice use a different exploration strategy, possibly due to a failing working memory or an attention deficit.

Further behavioral differences between *Pum2^XE772^* and wildtype mice were seen in the Morris Water Maze. Mice of both genotypes spent the same period of time in the target quadrant after the platform was removed, suggesting that they had normally functioning spatial memory. However, *Pum2^XE772^* mice entered the target quadrant significantly earlier and more frequently, suggesting enhanced memory or an increase in exploration motivation.

We also observed that *Pum2^XE772^* displayed a difference in nesting behavior. Nesting, a species-typical behavior is commonly used as a method to test for hippocampal dysfunction [25]. While there was already an observable trend in abnormal nesting behavior under normal conditions, this effect was much greater after a major experimental intervention. The fact that all but one *Pum2^XE772^*mouse left the Nestlet untouched after undergoing surgery for implantation of the EEG electrode, whereas 90% of wildtype mice built a perfect nest suggests that *Pum2*-deficient mice cope differently with environmental changes. An abnormal stress response could account for such differences but CORT analysis suggests that *Pum2^XE772^* mice are not more stressed *per se*. Impairment of nesting has been described in models of schizophrenia as a sign of self-neglect [26], as well as in models for Alzheimer's disease [27] and in Ts65Dn mice, a mouse model for Down syndrome [16]. Analogous to the *Pum*-deficient mice, there was a small difference in nesting behavior in Ts65Dn mice under normal conditions, whereas after profound environmental changes, nesting was significantly impaired, but not in diploid controls. Several mouse models for schizophrenia have also been described as being hyperactive and traveling longer distance in the activity chamber compared to wildtype mice [26], [28], [29]; we observed a similar behavioral pattern in *Pum2^XE772^* mice.

The finding in our previous report that *Pum2^XE772^* mice have smaller testes than wildtype mice [11] could imply the possibility of changes in the level of sex hormones like testosterone [30]. However, in the present study, both female and male mice had decreased body mass, and there were no sign of increased aggression, anxiety or abnormal fertility, which would be signs of steroid hormones changes [31].

An alternative explanation for the abnormal behavior of *Pum2^XE772^* mice derives from studies of mice with fragile X syndrome (FXS). FXS is caused by mutations in the *Fmr1* and/or *Fxr2* gene, the autosomal homolog of *Fmr1*, and is characterized by mental retardation and occasionally autism and epilepsy. Interestingly, Fmr1 protein, FMRP, has a similar function to Pumilio and has been shown to regulate translation and transport of a variety of mRNAs in the brain. Some of the potential Fmr1 targets have also been identified as putative Pum2 targets, including Psd95 in *Drosophila*, as well as App and Pnn in humans [9], [10], [32]–[34]. Given that both Fmr1 and Fxr2 proteins bind to human Pum1 [10] and that Pum2 mRNA was identified in a screen as a potential mouse Fmr1 target [35], it is possible that Pumilio and Fmr1 might also regulate each other. Moreover, Fmr1 and Pum2 are both recruited into miRNA complexes [10], [36], are involved in RNA localization and synapse morphology [37], [14], and both proteins are part of stress granules in neurons [38], [13], suggesting that Fmr1 and Pum2 might work together to regulate neurological processes in the brain. However, further analysis is needed to confirm this hypothesis.

Our findings reveal several similarities between Fmr1 knockout and *Pum2^XE772^* mice such as hyperactivity and likelihood of epileptic seizures. Other observations regarding the behavioral phenotype of *Pum2^XE772^* mice could suggest autistic features (decreased nest building, abnormal behavior in the water maze and NOR), although further tests are required to confirm this notion. Altogether, the behavioral changes seen in *Pum2^XE772^* mice and the similarities with other genetic models suggest that *Pum2* encodes a major brain factor that facilitates adaptation to environmental changes and challenges. The results presented here suggest that deficiency of *Pum2* prevents animals from adapting to overwhelming conditions and indicate that this gene could potentially be of importance in neuropsychiatric conditions.

**Figure 7:**
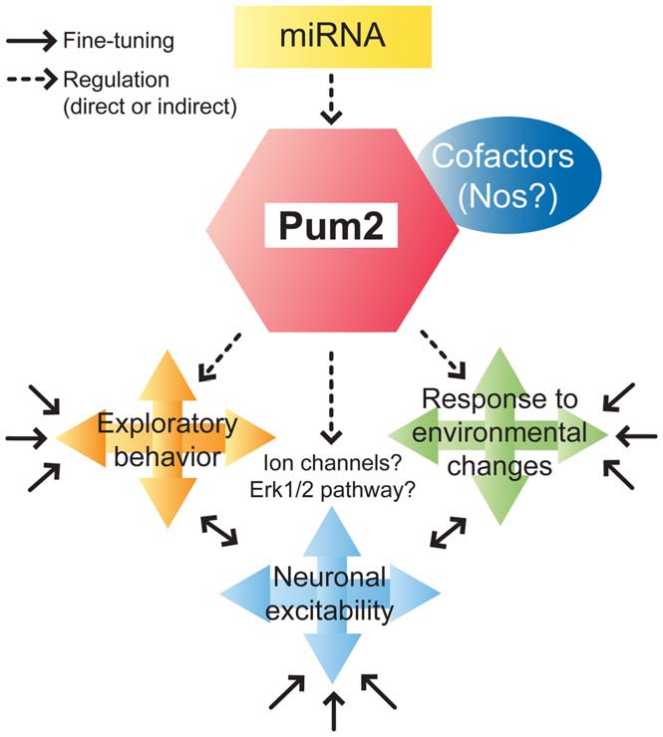
Hypothetical model of *Pum2* function in the mouse CNS. *Pum2*-mediated fine-tuning of regulatory pathways may affect neuronal excitability and other neural functions. This may be achieved by modifying the expression of ion channels. *Pum2* function, in turn, is thought to be fine-tuned by miRNAs [10], [15] and potentially needs co-factors to be effective.

Finally, we showed that *Pum2^XE772^* mice have spontaneous seizures and are more susceptible to seizure-inducing drugs. The fact that Pum2 potentially regulates a variety of genes in the brain makes it difficult to predict a single cause of the seizures. Also, if Pum2 were a major regulator in neuronal signaling, we would expect a more pronounced neuronal phenotype in *Pum2*-deficient mice. Therefore, it is most likely that Pum2 is responsible for fine-tuning regulatory pathways that are involved in neuronal excitability and other functions in the brain (Fig. 7). One possibility could be that Pumilio is involved in the regulation of ion channels as in *Drosophila*, where Pum regulates the voltage-gated sodium channel, *para*. Our gene expression analysis not only identified differential expression of genes in *Pum2^XE772^* mice suggested to be regulated by Pumilio [9], [10], [39], but also genes known to be involved in seizures and/or epilepsy (Hspbap1, Stat9, Bax, Mapk14, Nav1.2, Nav1.6). However, we only looked at a distinct subset of genes and the majority of changes in gene expression were relatively small. Since Pumilio only inhibits mRNA translation and does not necessarily target the mRNA for degradation, we expected modest changes in mRNA levels. Detailed protein analysis and a better understanding of mammalian Pumilio targets will be required to identify the precise mechanism that leads to the neuronal phenotype associated with *Pum2^XE772^* mice. Nevertheless, our findings demonstrate that *Pum2*-deficiency leads to a variety of neurobehavioral defects, including spontaneous seizures, increased susceptibility to seizure-inducing drugs and abnormal neuropsychiatric conditions, which may have implications for neurological diseases such as Parkinson's, epilepsy and autism.

## Materials and Methods

### Mouse husbandry, breeding and genotyping

All procedures involving mice were approved by the Animal Care and Use Committee of Stanford University. *Pum2^XE772^* mice are available for distribution at the Mutant Mouse Regional Resource Center, UC Davis (strain B6.129P2-*Pum2^Gt(XE772)Byg^*/Mmcd). Mice were kept in compatible breeding pairs and offspring was weaned at 21–28 days. Tail tip genotyping was performed as described [11]. For the monitoring of the body weight, mice were weighed at 3 weeks age and every two weeks thereafter. Altogether more than 600 mice were included in the weight monitoring and each time point was calculated using at least 10 animals (average N = 30) per sex and genotype.

### Transcardial perfusion

For transcardial perfusion animals were anesthetized with Avertin (Sigma-Aldrich, 400 mg/kg body weight). The chest was opened and the right atrium was cut open with scissors. A 21-gauge butterfly infusion needle was inserted into the left ventricle. Animals were transcardially perfused through the ventricular catheter with 40 ml PBS followed by 25 ml fixative (4% PFA in PBS). After perfusion the brain was removed, cryopreserved in increasing concentrations of sucrose in PBS (10%, 20%, 30%), embedded in O.C.T., flashfrozen and cryosectioned.

### Behavioral assessment

Behavior of mice was tested using standard behavioral assessment methods. All experiments were performed at the Stanford Behavioral and Functional Neuroscience Laboratory. All mice were male and between 11–16 weeks of age. We first performed the tests with a smaller group of animals (N = 6 wildtype/12 heterozygous/7 *Pum2^XE772^* mice). The tests that revealed any significant differences between wildtype and *Pum2^XE772^* mice (Activity chamber, Novel object recognition and Morris water maze) were repeated with a second larger cohort of mice (N = 17 WT/12 het/18 *Pum2^XE772^* mice).

The Activity chamber was used to track total distance moved, velocity, resting time and time spent in defined areas to quantify overall locomotor activity. The mice were habituated to the environment over several days. The mice were placed in the center of a square arena (43.2×43.2 cm) mounted with three planes of infrared detectors, within a specially designed sound-attenuating chamber (66×55.9×55.9 cm) and allowed to freely move for 10 minutes while being tracked by the automated tracking system. At the conclusion of each trial the surface of the arena was cleaned with 70% ethanol.

The Open Field test was performed in the arena of a white squared box (76×76 cm). Each animal was placed in the center of the open field arena and allowed to move freely for 10 minutes while being tracked by an automated tracking system. Time spent in different zones (center, periphery, border and corners; Fig. S2, G), velocity, and distance traveled were recorded. At the conclusion of each trial the surface of the arena was cleaned with 70% ethanol.

The Novel Object Recognition (NOR) test was conducted in an open field arena (48×38×27 cm). The two objects used, a golf ball and a pencil sharpener, were consistent in height and volume, but different in shape and appearance. Mice were habituated to the arena for 10 minutes for 3 days prior to data acquisition with two identical objects placed diagonally across from each other. On training day, animals were recorded while exploring the arena with the same objects for 10 minutes (2-trial-NOR) or 4 times for 3 minutes each (5-trial-NOR). Twenty-four hours after training, recording was repeated with one object replaced by a novel one (testing day). The amount of time spent exploring each object (nose sniffing and head orientation within <1.0 cm) was recorded. Between each trial, the open field arena and the objects were cleaned with 70% alcohol to eliminate olfactory traces.

Spatial memory testing was conducted using the Morris water maze (MWM) task. The test was performed in a tank (176 cm in diameter) filled with water at a temperature of 22.0±1.5°C. The water was made opaque with non-toxic tempera paint. A submerged platform (17 cm in diameter) was placed about 1–2 cm below the water surface. The water tank was surrounded by privacy blinds with four visual cues on the blinds. The mice were monitored by a video tracking system positioned directly above the tank and parameters such as escape latency, thigmotaxis, distance moved and velocity were recorded using the Noldus Ethovision software. During Hidden Platform training (HPT) the platform was positioned in one of the quadrant in the tank. Animals were released into the tank (drop location changed based on experimental setup) and were given a maximum of 90 seconds to find the submerged platform. The trial ended when the subject stayed on the platform for 3 seconds or when the duration of the trial expired. Each mouse went through 4 training days with 4 trials each day, separated by a 30-minute inter-trial-interval.

### Nesting behavior

Nesting behavior was assessed according to [17]. For baseline assessment 9–12 week old male mice (N = 10 wildtype and 10 *Pum2^XE772^* mice) that previously had been group-housed were placed in individual cages about 30 minutes before dark. Each animal was provided with a 2.7 g Nestlet. The next morning remaining Nestlets were weighed and nests were scored on a scale from 1–5 (1: Nestlet not touched or >90% intact, 2: Nestlet 50–90% intact; 3: Nestlet 50–90% shredded, 4: Nestlet >90% shredded, flat nest within ¼ of the cage, 5: (near) perfect nest with walls higher than the mouse for >50% of its circumference. Pictures were taken of all nests. For nesting assessment after a major environmental change, mice that had undergone surgery for EEG electrode implanting were group housed for 7 days and then placed in individual cages containing one Nestlet each (N = 8 wildtype and 8 *Pum2^XE772^* mice). Nests were scored 12 hours later.

### Corticosterone extraction and enzyme immunoassay

Corticosterone levels were assayed at base level from feces and under stress conditions from serum. Corticosterone was isolated from feces according to [40]. Briefly, mice were placed in a clean cage and all feces were collected after 8 hours. Feces was ground, dried at 37°C and frozen. Hormones were extracted from 100 mg aliquots in 1 ml 80% Methanol. To measure corticosterone levels under stress mice were restrained by placing them in 50 ml conical tubes with air holes in the walls for ventilation. After 20 minutes in the tube, the mice were euthanized, trunk blood was collected, allowed to clot, and red blood cells were separated from serum by centrifugation at 4°C. Sera were stored at −80°C until corticosterone level assay was performed. Corticosterone was quantified using the commercially available Correlate-EIA Corticosterone kit (Assay Designs). (N = 8 wildtype and 8 *Pum2^XE772^* mice).

### EEG recordings and seizure induction

Surgical preparation occurred under anesthesia delivered intraperitoneally with a xylazine/ketamine mix (10 and 3 mg/kg respectively). Three-month-old male *Pum2^XE772^* mice (N = 8) and wildtype littermates (N = 8) were implanted with two EEG electrodes (golden plated screws, 0.8 mm diameter). The position of the EEG was epidural, 1 mm lateral to bregma, and +1 mm frontal or −2 mm posterior to bregma. Electrodes were anchored to the skull and a connector using Metabond™ (Parkell Inc.) and acrylic dental cement. Mice were allowed 2 weeks of recovery from surgery and habituation to experimental conditions before the experiments (see [41] for a detailed protocol).

Recordings were achieved in 16 mice simultaneously using commercial hardware (EMBLA™) and software (Somnologica-3™) under standard conditions (individual cages, LD 12/12, food and water ad libitum, 24°C). Baseline recordings were achieved for 48 continuous hours prior the seizure induction experiments to ensure signal quality. Continuous EEG recordings were made throughout the seizure induction experiments comprising several minutes of a control period under saline s.c., followed immediately by the seizure inducing drug injection. Moreover, the mice were in continuous visual monitoring.

Seizure induction was achieved in two separate experiments using the chemoconvulsive agent, pentylenetetrazole (PTZ, Sigma-Aldrich). Sub-threshold PTZ injections: According to literature and our pilot studies the mice were first treated with 10 mg/kg PTZ followed by a second dose 10 mg/kg PTZ 15 minutes after the first. Above-threshold PTZ injections: 8 days after the previous experiment, mice were injected with 45 mg/kg PTZ.

EEG signals were amplified, filtered, and analog-to-digital converted at 2000 Hz, subsequently down-sampled and stored at 200 Hz.

The analysis of seizure activity was achieved using both behavioral assessment and EEG inspection. Behavior and EEG were scored by two different observers who were blind to the genotype. Abnormal behavior was scored according to [42]. Concomitantly, the occurrence of abnormal EEG patterns (high amplitude 5 to 10 Hz frequencies, and fast frequencies) was quantified by visual inspection throughout the recording session. Furthermore, for normalization and illustration purposes, spectral analysis of the EEGs was computed using the software Phytools™ and a Discrete Fourier Transform, yielding power spectra between 0 and 90 Hz (0.25 Hz resolution) using a 3-s window.

### Tissue collection, RNA Isolation and reverse transcription

For gene expression analysis in the mouse hippocampus, mice were decapitated and the brain quickly removed. The brains were sectioned in 500 µm sections with a Vibratome 1500 in ice-cold PBS. CA1, CA3 and DG were manually dissected and snapfrozen in liquid nitrogen. Total RNA from dissected brain tissue was extracted using the PicoPure kit (Molecular Devices). Briefly, 25 µl XB buffer was added to 2 pieces of frozen tissue, vortexed and incubated at 42°C for 30 min. After brief centrifugation the supernatant was used for RNA extraction according to the Picopure protocol with an additional DNase (Qiagen) treatment step. RNA was eluted in 30 µl elution buffer.

### Quantitative RT-PCR

To identify and quantify mRNA transcripts, 20 ng RNA was used in a combined reverse transcription and pre-amplification step (CellsDirect One-step qRT-PCR kit, Invitrogen) with a set of 96 Taqman assays (Applied Biosystems, Table S1) with following PCR protocol: 50°C, 15 min; 70°C, 2 min; 18 cycles of 95°C, 15 s; 60°C, 4 min. Pre-amplified cDNA was diluted 1∶3 in TE, loaded onto a 96.96 Dynamic Array chip (Fluidigm) and run on the Fluidigm Biomark System according to the manufacturer's protocol. All reactions were run as duplicates on each chip. Raw Ct values were imported into qBasePlus (Biogazelle). All duplicates were averaged or, when more than 1.0 Ct different from each other, excluded from the analysis. Ct values were normalized to three reference genes (Eno2, Syp, Tubb3 for neuronal genes and Ctnnb1, Gapdh, Ubc for all others). Reference gene stability (M) was confirmed with the geNorm algorithm. In one run the stability value for Tubb3 was above 0.5 and only Eno2 and Syp were used for normalization. All values were normalized to the average and exported into Excel for statistical analysis.

### Statistical analysis

All tests were calculated using Graphpad Prism software. For most experiments, groups were compared using two-tailed, two-sampled *t*-test. For the activity chamber we used repeated measures two-way ANOVA to analyze interaction of genotype and time. The Mann Whitney test was used for EEG analysis. For gene expression analysis, all 4 technical replicates for each sample were averaged and checked for outliers (2-fold standard deviation), which were excluded from the analysis. Biological replicates (N = 6–7 for wildtype and 8–9 for *Pum2^XE772^*) were averaged and outliers (2-fold standard deviation) were removed. For each gene the *Pum2^XE772^* value was normalized to the wildtype value. Mean values of the two genotypes were compared using two-tailed, two-sampled *t*-test.

## Supporting Information

Table S1 List of all Taqman assays that were used for gene expression analysis.
(PDF)

Figure S1 Representative histology images. Brains of wildtype (left panels) and *Pum2^XE772^* mice were stained with antibodies against NeuN (top), Neurofilament (middle) and GFAP (bottom).
(TIF)

Figure S2 Open Field Test. (A) *Pum2^XE772^* mice travel a significantly longer distance compared to wildtype mice. (B) *Pum2^XE772^* mice travel with a significantly higher velocity than wildtype mice. (C) Frequency of rearing is not significantly different between the two genotypes. (D) Mice of both genotypes spend most of the time in the border area and avoid the center area. (E) In accordance with longer distance and higher velocity, *Pum2^XE772^* mice enter all areas at a higher frequency than wildtype mice. (F) There is no significant difference in latency of first entry into any of the areas after being place in the center between the two genotypes. (G) Scheme of the areas in the Open Field Test. (*p<0.05).
(TIF)

Figure S3 Five Trial NOR. (A–B) During the five training sessions, wildtype (A) and *Pum2^XE772^* (B) mice spend equal time exploring each of the two object objects. (C) Mice of both genotypes memorize a known object and spend more time with a novel object. (D) The discrimination index shows that mice of both genotypes recognize the novel object.
(TIF)

Movie S1 Type 5 seizure with simultaneous EEG trace of wildtype mouse in 45 mg/kg PTZ seizure screen.
(MOV)

Movie S2 Type 5 seizure with simultaneous EEG trace of *Pum2^XE772^* mouse in 45 mg/kg PTZ seizure screen.
(MOV)

Movie S3 Five wildtype mice with type 5 seizures in 45 mg/kg PTZ seizure screen.
(MOV)

Movie S4 Five *Pum2^XE772^* mice with type 5 seizures in 45 mg/kg PTZ seizure screen.
(MOV)
