# Supplementary material for: Pumilio-2 Function in the Mouse Nervous System
Source: PLoS One. 2011 Oct 7;6(10):e25932. doi: 10.1371/journal.pone.0025932 (PMC3189250; doi:10.1371/journal.pone.0025932)
Supplement: Table S1 — List of all Taqman assays that were used for gene expression analysis. (PDF) [file pone.0025932.s001.pdf]

Category	Symbol	RefSeq	Taqman assay ID	Alias	Gene name
**Neuronal**	Ache	NM_009599.3	Mm00477275_m1		Acetylcholinesterase
	App	NM_007471.2	Mm00431827_m1		amyloid beta (A4) precursor protein
	Dlg1	NM_007862.2	Mm00492189_m1	Sap97	discs, large homolog 1
	Dlg2	NM_011807.2	Mm00457160_m1	PSD93	discs, large homolog 2
	Dlg3	NM_016747.2	Mm00497394_m1	SAP102	discs, large homolog 3
	Dlg4	NM_001109752.1	Mm00492193_m1	PSD95	discs, large homolog 4
	Kcnq1	NM_008434.2	Mm00434638_m1	Kcna9	potassium voltage-gated channel, subfamily Q, member 1
	Kcnq2	NM_001003825.2	Mm00440084_mH		potassium voltage-gated channel, subfamily Q, member 2
	Kcnq3	NM_152923.1	Mm00548884_m1		potassium voltage-gated channel, subfamily Q, member 3
	Merg1a	NM_013569.2	Mm00465370_m1	Kcnh2	potassium voltage-gated channel, subfamily H (eag-related), member 2
	Nav1.1	NM_018733.2	Mm01329044_mH	Scn1a	sodium channel, voltage-gated, type I, alpha
	Nav1.2	NM_001099298.2	Mm01270369_m1	Scn2a1	sodium channel, voltage-gated, type II, alpha 1
	Nav1.5	NM_021544.3	Mm00451973_mH	Scn5a	sodium channel, voltage-gated, type V, alpha
	Nav1.6	NM_001077499.1	Mm01300412_mH	Scn8a	sodium channel, voltage-gated, type VIII, alpha
	Ncam1	NM_010875.3	Mm00456815_m1		Neural cell adhesion molecule
	Nse	NM_013509.2	Mm00469062_m1	Eno2	enolase 2, gamma neuronal
	Syn	NM_009305.2	Mm00436850_m1	Syp	synaptophysin
	Tubb3	NM_023279.2	Mm00727586_s1		tubulin, beta 3
	Zic3	NM_009575.2	Mm00494362_m1	Bn	zinc finger protein of the cerebellum 3
**RNA and Translational Regulation**	Ago2	NM_153178.3	Mm00838341_m1	Eif2c2	eukaryotic translation initiation factor 2C, 2
	Ago3	NM_153402.2	Mm01188534_m1	Eif2c3	eukaryotic translation initiation factor 2C, 3
	Cpsf2	NM_016856.3	Mm00489745_m1		cleavage and polyadenylation specific factor 2
	Ddx20	NM_017397.3	Mm00600300_g1	Gemin3	DEAD (Asp-Glu-Ala-Asp) box polypeptide 20
	Dicer	NM_148948.2	Mm00521731_m1		Dicer1, Dcr-1 homolog (Drosophila)
	Eif1a	NM_010120.5	Mm00456651_m1		eukaryotic translation initiation factor 1A
	Eif3b	NM_133916.2	Mm00659801_m1		eukaryotic translation initiation factor 3, subunit B
	Eif3c	NM_146200.1	Mm01278697_m1	NipilA3	eukaryotic translation initiation factor 3, subunit C
	Eif3e	NM_008388.2	Mm01700222_g1		eukaryotic translation initiation factor 3, subunit E
	Eif4e	NM_007917.3	Mm00725633_s1		eukaryotic translation initiation factor 4E
	Eif5b	NM_198303.2	Mm01227234_m1		eukaryotic translation initiation factor 5B
	Fmr1	NM_008031.2	Mm00484415_m1	Fmrp	fragile X mental retardation syndrome 1 homolog
	Fxr2	NM_011814.2	Mm00839957_m1		fragile X mental retardation, autosomal homolog 2
	Gemin5	NM_172558.2	Mm00553372_m1		gem (nuclear organelle) associated protein 5
	Nanos1	NM_178421.3	Mm02525647_s1	Nos1	nanos homolog 1 (Drosophila)
	Nanos2	NM_194064.2	Mm02525720_s1	Nos2	nanos homolog 2 (Drosophila)
	Paip1	NM_145457.3	Mm00520884_m1		polyadenylate binding protein-interacting protein 1
	Papola	NM_011112.3	Mm01334253_m1	Pap; Plap	poly (A) polymerase alpha
	Piwil2	NM_021308.1	Mm00502383_m1	Mili	piwi-like homolog 2
	Prpf4b	NM_013830.2	Mm00443401_m1	Prp4k	PRP4 pre-mRNA processing factor 4 homolog B
	Pum1	NM_030722.2	Mm00472886_m1		pumilio 1 (Drosophila)
	Pum2	NM_030723.2	Mm00472902_m1		pumilio 2 (Drosophila)
	Pum2-2	NM_030723.2	Mm01270654_m1		pumilio 2 (Drosophila)
	Sip1	NM_025656.4	Mm00491823_m1	Gemin2	survival of motor neuron protein interacting protein 1
	Syncrip	NM_019796.4	Mm00479927_m1	Nsap1	synaptotagmin binding, cytoplasmic RNA interacting protein

Category	Symbol	RefSeq	Taqman assay ID	Alias	Gene name
DNA and Transcriptional Regulation	Brg1	NM_011417.2	Mm01151944_m1	Smarca4	SWI/SNF related, matrix associated, actin dependent regulator of chromatin, subfamily a, member 4
	Dnmt1	NM_010066.3	Mm00599763_m1		DNA methyltransferase (cytosine-5) 1
	Dnmt3a	NM_007872.4	Mm00432870_m1		DNA methyltransferase 3A
	Dnmt3b	NM_001003960.3	Mm01240113_m1		DNA methyltransferase 3B
	Gtf2f1	NM_133801.2	Mm00505439_m1	TfIIAa/b	general transcription factor II A, 1
	Hdac2	NM_008229.2	Mm00515108_m1		histone deacetylase 2
	Hsf1	NM_008296.2	Mm01201402_m1		heat shock factor 1
	Hsp70.1	NM_010478.2	Mm03038954_s1	Hspa1b	heat shock protein 1B
	Hspbap1	NM_175111.3	Mm00613037_m1		Hspb (heat shock 27kDa) associated protein 1
	Id3	NM_008321.2	Mm00492575_m1		inhibitor of DNA binding 3
	Nfat5	NM_133957.3	Mm00467257_m1	TonEBP	nuclear factor of activated T-cells 5
	Pax6	NM_013627.4	Mm00443072_m1		paired box gene 6
	Sin3b	NM_009188.3	Mm01247119_m1		transcriptional regulator, SIN3B
	Sox9	NM_011448.4	Mm00448840_m1		SRY-box containing gene 9
	Stat3	NM_213659.2	Mm01219775_m1	Aprf	signal transducer and activator of transcription 3
Cell Cycle	Ccnb1	NM_172301.3	Mm03053893_gH		Cyclin B1
	Ccne	NM_007633.2	Mm01266311_m1		Cyclin E1
	Cdc25a	NM_007658.3	Mm00483166_m1		cell division cycle 25 homolog A
	Cdc2a	NM_007659.3	Mm00772471_m1	Cdk1	cell division cycle 2 homolog A
	Cdkn1b	NM_009875.4	Mm00438168_m1	p27Kip1	cyclin-dependent kinase inhibitor 1B
	Pard3	NM_033620.2	Mm00473929_m1	Par3	partitioning defective 3 homolog
	Pard6a	NM_001047435.1	Mm00480004_m1	Par6	partitioning defective 6, homolog alpha
Apop-tosis	Bax	NM_007527.3	Mm00432050_m1		Bcl2-associated X protein
	Bcl2	NM_009741.3	Mm00477631_m1		B-cell leukemia/lymphoma 2
	Becn1	NM_019584.3	Mm00517174_m1		beclin 1, autophagy related
Other	Bmpr1a	NM_009758.4	Mm00477650_m1		bone morphogenetic protein receptor, type 1A
	Gnb2l1	NM_008143.3	Mm00515010_m1	Rack1	guanine nucleotide binding protein (G protein), beta polypeptide 2 like 1
	Ifitm3	NM_025378.2	Mm00847057_s1	Fragilis	interferon induced transmembrane protein 3
	Kdr	NM_010612.2	Mm00440111_m1	VEGFR2, Flk1	kinase insert domain protein receptor
	Kitl	NM_013598.2	Mm00442972_m1	SCF, SF	Kit ligand
	MapK1	NM_001038663.1	Mm00442479_m1	Erk2	mitogen-activated protein kinase 1
	Mapk1-l	NM_011949.3	Mm00466437_s1	Erk2	mitogen-activated protein kinase 1
	MapK14	NM_011951.2	Mm00442497_m1	p38alpha	mitogen-activated protein kinase 14
	Rbbp6	NM_011247.2	Mm00656624_m1	P2P-R	retinoblastoma binding protein 6
	Sparc	NM_009242.4	Mm00486332_m1	BM-40	secreted acidic cysteine rich glycoprotein
	Vegfa	NM_001025250.3	Mm00437306_m1		vascular endothelial growth factor A
House-keeping	Ctnnb1	NM_007614.2	Mm01350394_m1	Catnb	catenin (cadherin associated protein), beta 1
	Gapdh	NM_008084.2	Mm99999915_g1		glyceraldehyde-3-phosphate dehydrogenase
	Ubc	BC008661.1	Mm01201237_m1		ubiquitin C
