# Supplementary figures and images for: Pumilio-2 Function in the Mouse Nervous System
Source: PLoS One. 2011 Oct 7;6(10):e25932. doi: 10.1371/journal.pone.0025932 (PMC3189250; doi:10.1371/journal.pone.0025932)

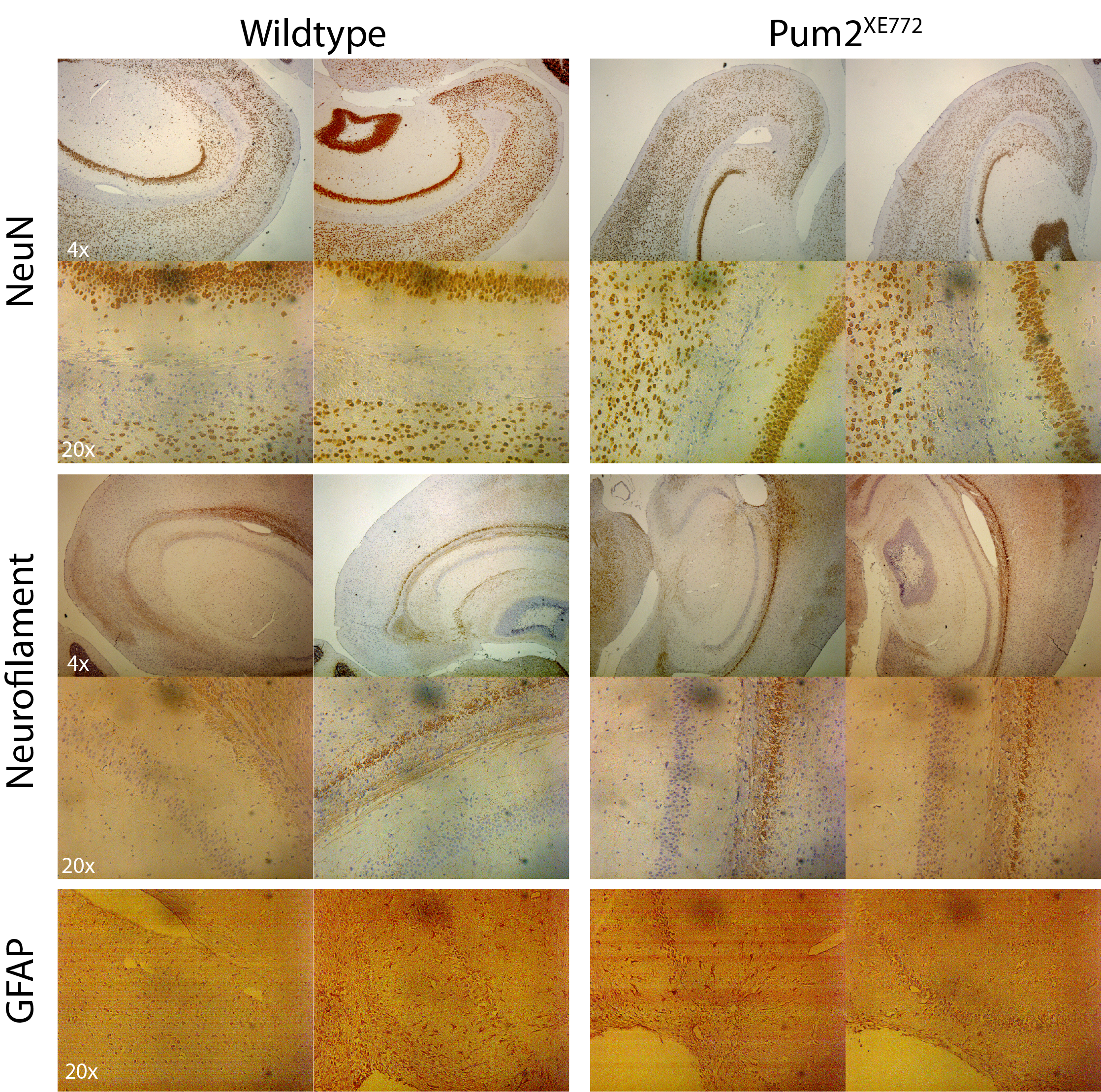

Supplement: Figure S1 — Representative histology images. Brains of wildtype (left panels) and Pum2XE772 mice were stained with antibodies against NeuN (top), Neurofilament (middle) and GFAP (bottom). (TIF) [file pone.0025932.s002.tif]

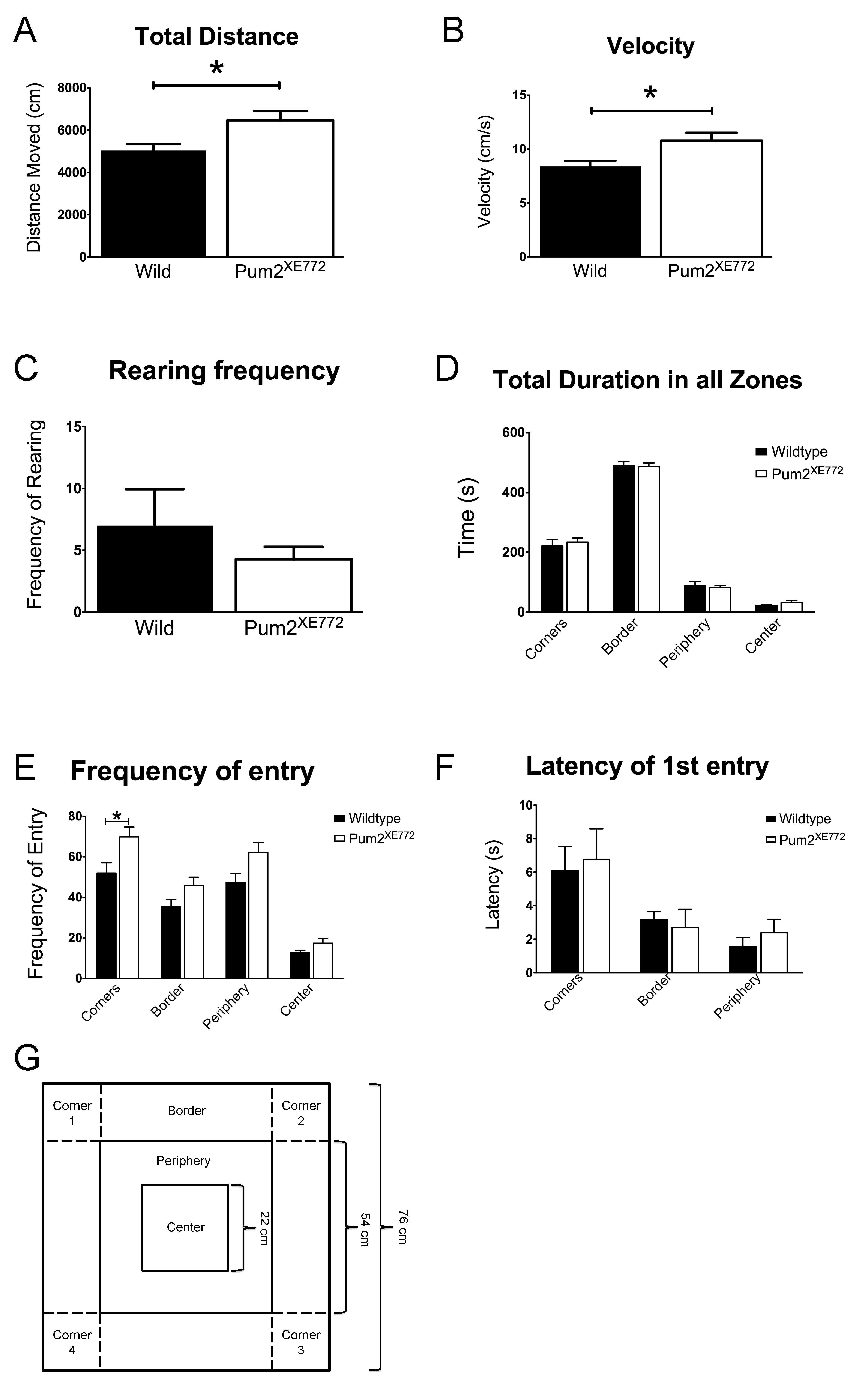

Supplement: Figure S2 — Open Field Test. (A) Pum2XE772 mice travel a significantly longer distance compared to wildtype mice. (B) Pum2XE772 mice travel with a significantly higher velocity than wildtype mice. (C) Frequency of rearing is not significantly different between the two genotypes. (D) Mice of both genotypes spend most of the time in the border area and avoid the center area. (E) In accordance with longer distance and higher velocity, Pum2XE772 mice enter all areas at a higher frequency than wildtype mice. (F) There is no significant difference in latency of first entry into any of the areas after being place in the center between the two genotypes. (G) Scheme of the areas in the Open Field Test. (*p<0.05). (TIF) [file pone.0025932.s003.tif]

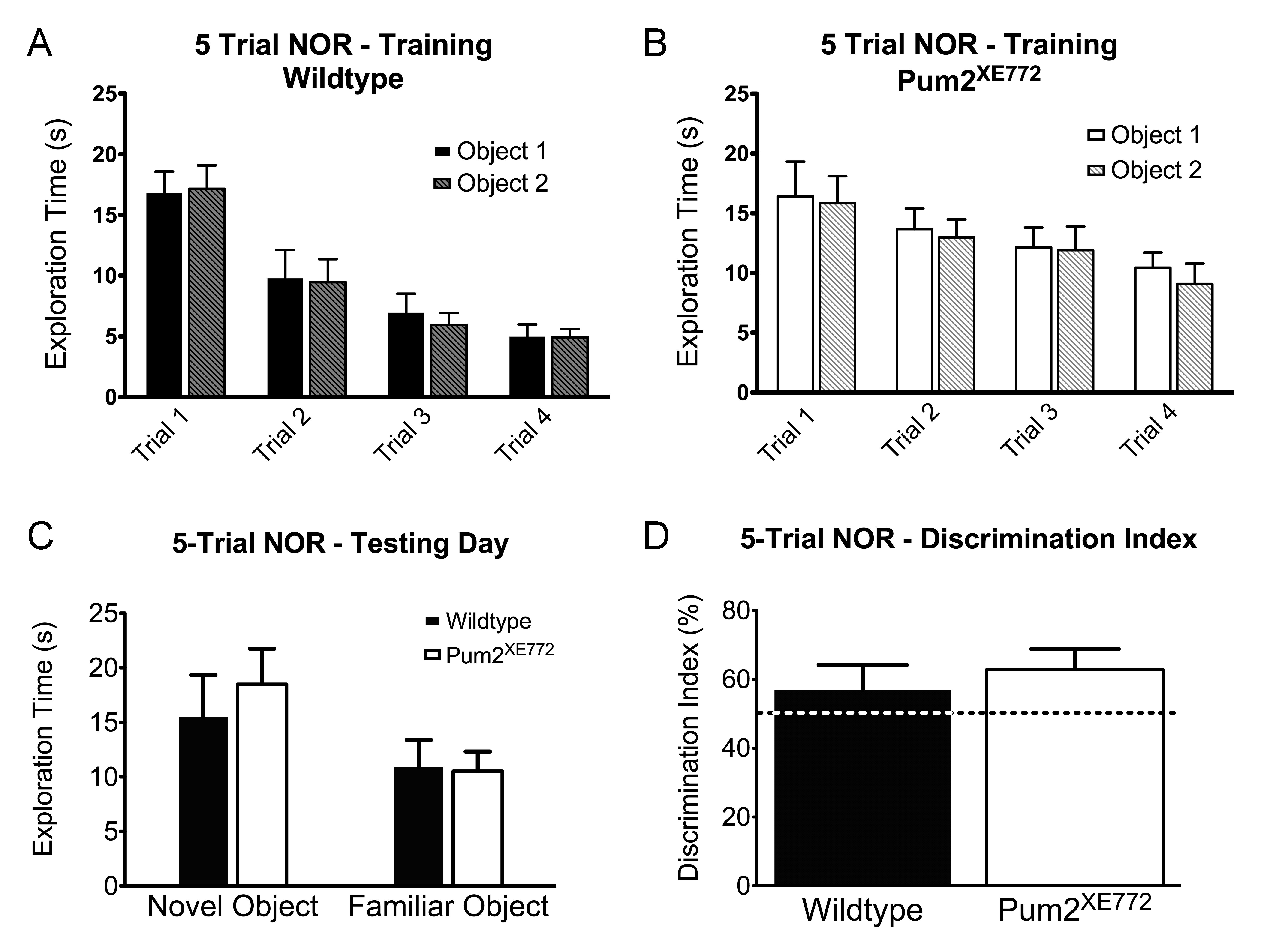

Supplement: Figure S3 — Five Trial NOR. (A–B) During the five training sessions, wildtype (A) and Pum2XE772 (B) mice spend equal time exploring each of the two object objects. (C) Mice of both genotypes memorize a known object and spend more time with a novel object. (D) The discrimination index shows that mice of both genotypes recognize the novel object. (TIF) [file pone.0025932.s004.tif]
